# Chylothorax in a child with rifampicin-resistant tuberculosis

**DOI:** 10.7196/SARJ.2019.v25i3.237

**Published:** 2019-09-17

**Authors:** B McLaren, X Song, E Mate, C Jardine, T Mabaso, V Mammen, S Lala, Z Dangor, C Verwey

**Affiliations:** Department of Paediatrics and Child Health, Faculty of Health Sciences, University of the Witwatersrand, and Chris Hani Baragwanath Academic Hospital, Johannesburg, South Africa

**Keywords:** drug resistant tuberculosis, tuberculosis, paediatric tuberculosis, endobronchial tuberculosis, chylothorax

## Abstract

Chylothorax is rare in children. Only a few cases of tuberculosis (TB)-associated chylothorax have been reported. We present a child on standard four-drug TB treatment who presented with wheezing and a chylothorax. Bronchoscopy showed caseating lymph nodes, and rifampicin-resistant TB was identified from the bronchoalveolar lavage specimen. There was marked clinical and radiological improvement 1 month after starting multidrug-resistant (MDR) TB treatment and steroids. The association of chylothorax and MDR-TB has not been described in children. MDR-TB should be considered in children who fail adherent, empirically started drug-susceptible TB treatment.

## Background

The burden of pulmonary tuberculosis (PTB) is very high in subSaharan Africa, with a reported incidence of 500 per 100 000 population.^[1]^ Children account for ~15% of the total tuberculosis (TB) burden in developing countries.^[2]^ Culture-confirmed TB accounts for only 10 - 15% of children treated for TB.^[3]^ This is a result of the paucibacillary nature of PTB in children, and the fact that specimens are often not obtained. Drug susceptibility testing can only be done if bacteriological confirmation is achieved. In settings with a high incidence of drug-resistant TB, drug resistance should be considered in children with clinically diagnosed TB who have not improved, or have progressively worsened, despite adequate first-line TB therapy. Although drug-resistant TB has not been reported to be more virulent than drug-susceptible TB,^[4]^ the delay in identifying and treating these children may result in more advanced or disseminated disease.

Chylothorax is defined as a pleural effusion with triglyceride levels >110 mg/dL and the presence of chylomicrons.^[5]^ It is hypothesised that TB lymph nodes erode through the thoracic duct, resulting in chylous fluid leaking into the thoracic space, of which only a few childhood cases have been reported. We describe a chylothorax in a child with rifampicin-resistant endobronchial TB.

## Case presentation

An HIV-exposed uninfected 20-month-old boy presented to Chris Hani Baragwanath Academic Hospital in Johannesburg, South Africa, with a 2-day history of shortness of breath, cough and noisy breathing. He had also experienced night sweats, fever and vomiting for a week. Two weeks prior to this presentation, he had been diagnosed with bacteriologically unconfirmed PTB at a primary healthcare clinic and was empirically started on a four-drug antituberculosis treatment regimen (rifampicin, isoniazid, pyrazinamide and ethambutol). The diagnosis was based on a chest X-ray (Fig. 1A) and a positive Mantoux test. His father (a household contact) had been diagnosed with PTB, and had been on first-line antituberculosis treatment for 4 months. The boy had had normal growth and development up to 1 year of age, but had subsequently lost weight, and fallen from a weight-for-height *z*-score +2 to the median over the preceding 8 months.

On admission, he was apyrexial, tachycardic and in moderate respiratory distress, with oxygen saturations of 97% in room air. He had marked chest wall indrawing and bilateral posterior cervical and axillary lymph nodes (<0.5 cm in diameter). He was clinically hyperinflated, with bilateral wheezing. The rest of his clinical examination was normal.

### Case management

He was diagnosed with viral bronchiolitis, and therefore a chest X-ray was not performed. He was started on oxygen and hypertonic saline nebulisation. He responded poorly to this initial treatment and was therefore started on oral corticosteroids and amoxicillin on day 2 of hospitalisation, and his first-line antituberculosis treatment was continued. Gastric aspirate samples for microscopy for acid-fast bacilli, Xpert MTB/RIF (Cepheid, USA) and culture for mycobacteria were negative.

During his hospital admission period his wheezing persisted, and he remained in moderate respiratory distress. On the fifth day of admission, he developed percussion dullness and absent breath sounds over the right hemithorax.

A chest X-ray at this point revealed a large right-sided pleural effusion (Fig. 1B). A computed tomography scan of the chest revealed hilar lymphadenopathy and a large right-sided pleural effusion. The thoracic duct could not be visualised. Diagnostic pleurocentesis confirmed a chylothorax. It was suspected that tuberculous lymph nodes had eroded the thoracic duct causing the chylothorax, and therefore a bronchoscopy was performed, which revealed caseating lymph nodes in the right main bronchus suggestive of TB, and 90% obstruction of the left main bronchus. The Xpert MTB/RIF (Cephaid, USA) test on a bronchoalveolar lavage specimen identified rifampicinresistant *Mycobacterium tuberculosis*. Gastric aspirates, pleural fluid and bronchoalveolar lavage samples were all smear-negative for acidfast bacilli and mycobacterial culture-negative.

### Treatment

The chylothorax was managed with a low-fat diet (with additional medium-chain triglycerides), therapeutic taps for worsening respiratory distress and an octreotide infusion. The boy was commenced on multidrug-resistant TB (MDR-TB) treatment (amikacin, levofloxicin, ethionamide, terizidone, pyrazinamide, ethambutol and high-dose isoniazid) and oral steroids.

### Outcomes and follow-up

**Fig. 2:**
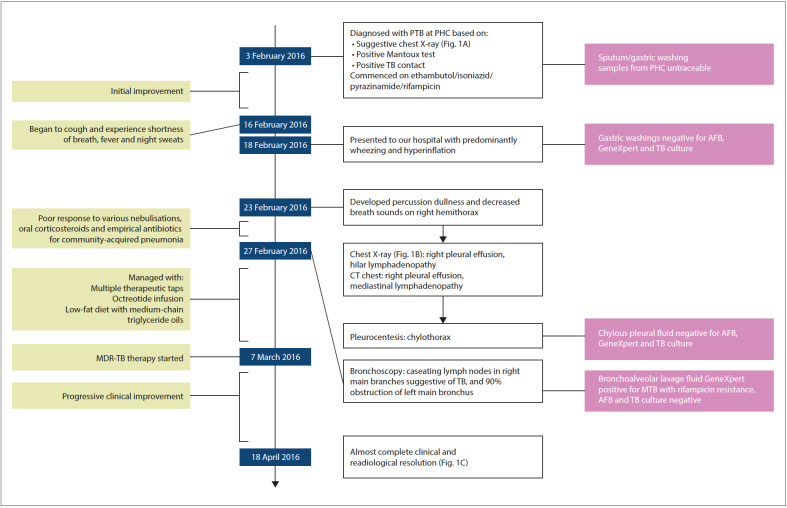
Case overview. MDR-TB = multidrug-resistant tuberculosis PTB = pulmonary tuberculosis PHC = primary healthcare clinic CT chest = computed tomography chest scan AFB = acid-fast bacilli MTB = *Mycobacterium tuberculosis*

For further management, he was transferred to a hospital dedicated to the care of MDR-TB patients. One month later, he showed marked clinical improvement, with almost complete radiological resolution of the chylothorax, and reduced hilar lymphadenopathy (Fig. 1C). The full case overview is shown in Fig. 2.

## Discussion

To our knowledge, this is the first reported case of a chylothorax in a child with drug-resistant PTB. We suspect that this child had had endobronchial PTB that had not responded to first-line TB therapy, and progressed to large airway compression and infiltration of the thoracic duct, resulting in chylous fluid leaking into the pleural space. Other possibilities to consider would be paradoxical enlargement of lymph nodes from partial TB treatment, as oral steroids were not initiated at the initial presentation to the primary healthcare clinic.^[6]^

In children, congenital malformations of the lymphatic system are the most common medical cause of chylothorax, although infrequent outside the neonatal period.^[5]^ Chylothorax due to malignancies is common in adults, but rare in children.^[5]^ TB-associated chylothorax has been described, but this is limited to case reports. A PubMed search using the MeSH terms ‘tuberculosis’ and ‘chylothorax’ identified seven case reports of children with TB-associated chylothorax (age of presentation ranging from 4 months to 17 years).^[7–12]^ In three of the cases, TB was bacteriologically confirmed on gastric aspirate samples, in one case on bronchioalveolar lavage and in two cases on pleural fluid. All cultures were susceptible to isoniazid and rifampicin. Most cases were associated with large TB perihilar lymphadenopathy. Our case differs in that the chylothorax was associated with drug-resistant TB. The delay in diagnosing drug-resistant TB and initiating appropriate second-line antituberculosis treatment may have led to progression of disease in this child, and this once again highlights the importance of vigorous contact screening, thorough clinical and microbiological investigation and initiation of appropriate antituberculosis treatment, as well as the implementation of a good follow-up plan for each case of TB.

## Conclusion

This case highlights the difficulties in managing children diagnosed with unconfirmed TB, and the fact that drug-resistant TB must be considered in children who do not respond to conventional first-line TB treatment.

Although endobronchial TB is common in children, severe disease resulting in bronchial obstruction and progression to invasion of other mediastinal structures such as the thoracic duct is infrequent.

## Figures and Tables

**Fig. 1A:**
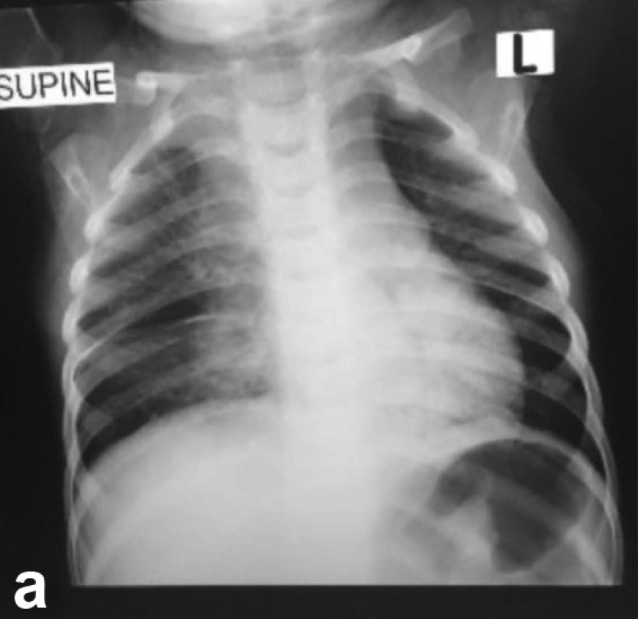
Chest X-ray on diagnosis of unconfirmed tuberculosis at the local clinic 1 month prior to index presentation, demonstrating attenuation of the trachea and right and left main bronchus.

**Fig. 1B:**
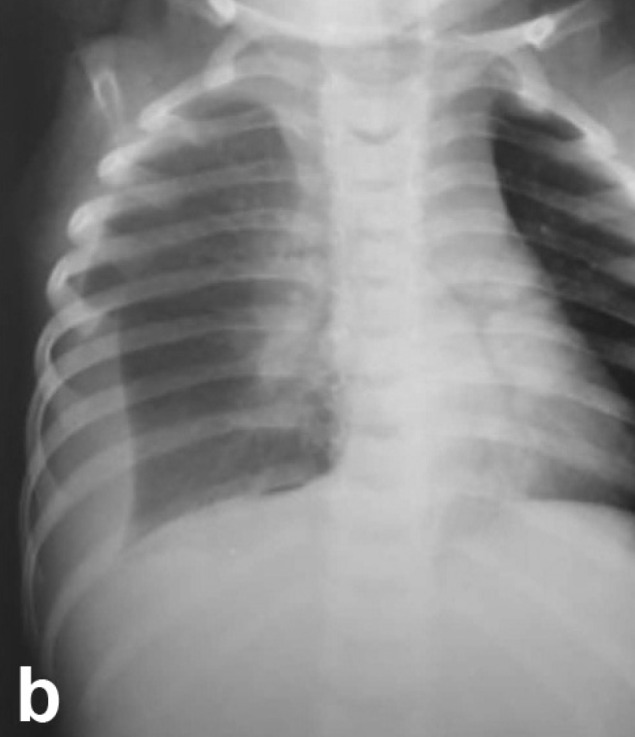
Chest X-ray on day 5 of admission, demonstrating right pleural effusion.

**Fig. 1C:**
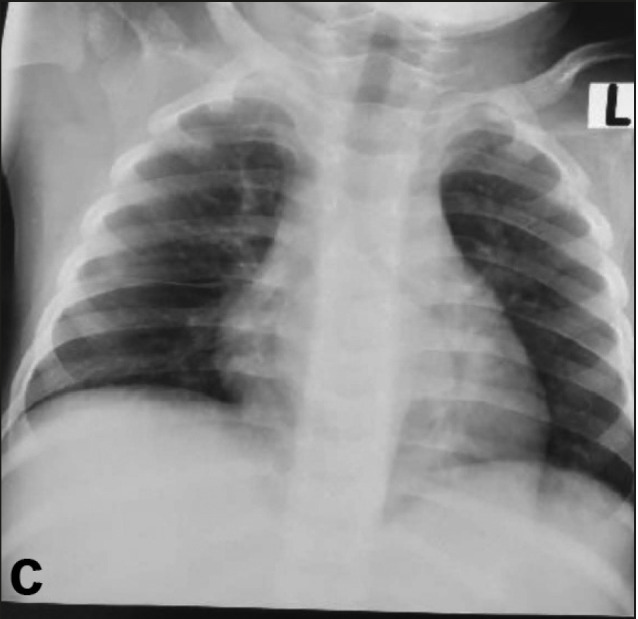
Chest X-ray after a month of rifampicin-resistant TB treatment, demonstrating radiological resolution of the chylothorax and loss of the attenuation of the trachea, right and left main bronchus.
